# Highly Sensitive Lateral Flow Immunoassay for Clenbuterol and Structurally Similar Beta2-Agonists in Meat

**DOI:** 10.3390/foods14233982

**Published:** 2025-11-21

**Authors:** Elena A. Zvereva, Olga D. Hendrickson, Dmitriy V. Sotnikov, Anatoly V. Zherdev, Xinxin Xu, Chuanlai Xu, Boris B. Dzantiev

**Affiliations:** 1 A.N. Bach Institute of Biochemistry, Research Center of Biotechnology of the Russian Academy of Sciences, Leninsky Prospect 33, Moscow 119071, Russia; zverevaea@yandex.ru (E.A.Z.); odhendrick@gmail.com (O.D.H.); sotnikov-d-i@mail.ru (D.V.S.); zherdev@inbi.ras.ru (A.V.Z.); 2 State Key Lab of Food Science and Technology, School of Food Science and Technology, Jiangnan University, Wuxi 214122, China

**Keywords:** growth promoters, clenbuterol, immunochromatography, gold nanoparticles, meat products, food safety

## Abstract

Beta-agonists are growth promoters sparking considerable interest in animal husbandry. However, their numerous negative effects on health have led to a number of restrictions on their presence in agricultural products, which differ depending on the type of preparation and food. In this regard, there is a demand for methods of their mass, rapid, and easy control with strict, focused selectivity. In this paper, a lateral flow immunoassay (LFIA) with clenbuterol (CLE), a priority β2-agonist for food safety, as the main target compound is proposed. The LFIA is based on indirect labeling of specific antibodies by gold nanoparticles via anti-species antibodies. The development of the LFIA involved optimizing the concentrations of immunoreagents and the composition of the reaction mixture (buffer type and pH, ionic strength, detergents, and additional components). In qualitative (visual) mode, the LFIA detects up to 1.0 ng/mL of CLE. In quantitative mode, the detection limit reaches 0.02 ng/mL, surpassing previously described colorimetric LFIAs. The selectivity of the obtained and used monoclonal antibodies allows for the group-specific detection of CLE and structurally close (the presence of a trimethyl residue, similar charge distribution in the benzene ring) common β2-agonists—salbutamol and mabuterol—distinguishing them from other β-agonists, including the widely used β1-agonist ractopamine, which differs in application and biological activity. The assay time is 15 min. The application of the LFIA for meat samples demonstrated that the CLE recovery ranged between 86% and 104%. The obtained results confirm the effectiveness and competitive potential of the developed assay for screening meat products outside of laboratories.

## 1. Introduction

Growth stimulants are popular tools used to intensify livestock production. Thus, the application of compounds belonging to β-adrenoreceptor agonists (β-agonists) leads to more efficient feed conversion and reduced costs in raising farm animals and poultry [1]. Beta-agonists stimulate specific receptors in muscle tissue, causing increased transformation of fat and synthesis of skeletal muscle protein, which leads to muscle fiber growth [2]. Due to this, the goal of unscrupulous producers is to obtain more edible muscle meat, for example, more tender (beef) or lean (pork) at lower costs [3]. However, the intake of β-agonists into the human body through foods disrupts a row of metabolic processes and negatively affects the cardiovascular and nervous systems [4]. Therefore, a number of legislative restrictions have been established that regulate the use of β-agonists and state requirements for their maximum permissible content in foodstuffs [2,5].

The chemical range of β-agonist drugs produced on a mass scale and used in medicine and animal husbandry is quite diverse [6]. They are classified based on the specific receptor they primarily target (β1, β2, or β3) and on the duration of their action (short-acting, long-acting, or ultra-long-acting). The action of β-agonists varies by species, depending on which type of β-receptors predominates as well as on the pharmacokinetics and pharmacodynamics of the β-agonist in that species. Therefore, safety demands for β-agonists as food contaminants depend on specific features of the target compound.

One of the common representatives of β-agonists is clenbuterol (CLE, (RS)-1-(4-amino-3,5-dichlorophenyl)-2-(tert-butylamino)ethan-1-ol)). CLE is characterized by high bioavailability when administered orally and is applied in medicine and veterinary due to its bronchiolitic and tocolytic effects. Clenbuterol is prohibited for growth promotion in animal husbandry in the EU, USA, China, and other countries, but cases of its illegal use are regularly revealed [2,7]. Therefore, sensitive and specific methods for detecting CLE in foodstuffs are in high demand.

The existing variety of methods includes chromatographic, electrochemical, and immunochemical methods [1,8,9,10]. The advantages of the latter are attributed to their implementation without expensive equipment and highly qualified personnel, simple sample preparation, and productive testing of large quantities of samples, as close as possible to the places of their collection. Of the analytical methods based on immune recognition, enzyme-linked immunosorbent assays (ELISAs) and immunochromatographic assays (LFIAs) are most in demand in modern practice as a result of the established industrial technologies for producing test systems. The application niche of ELISAs is determined by the possibility of productive quantitative assessment of the analyte content in a large number of samples in a few hours using a photometer. ELISAs for CLE have been successfully realized in several variants [11,12,13] and are available as commercial tests of Abbexa, IndiFOSS, Quicking Biotech, R-Biopharm, etc.

LFIA is based on the initiation of all interactions by contact of the sample and a multimembrane composite with pre-applied reagents. This eliminates the need for laboratory equipment for testing, significantly accelerating and simplifying the assay. In this way, the LFIA is ideally adopted for mass on-site screening control [14]. LFIAs have been developed for CLE detection using various nanodispersed labels [15,16,17,18,19,20,21,22].

The existing developments of immunochemical systems for detecting β-agonists are based on the use of different antibody preparations, which allow either highly selective detection of one compound or simultaneous monitoring of a number of β-agonists, depending on the practical problems being addressed. However, the described developments of polyspecific determination of β-agonists were aimed at finding solutions for detecting the widest possible range of compounds [15,23,24]. The task of differentiating groups within β-agonists that differ in biological action was not set in such studies. However, it is practically justified not to form a long (and expensive for users) panel of tests for each individual compound, but to distinguish toxic contaminants with different biological effects and degrees of danger.

Therefore, in this work, a lateral flow test system was developed for the differential control of β-agonists with simultaneous detection of CLE and several structurally close β2-agonists, but without interfering with the analysis of the known controlled contaminant β1-agonist ractopamine. It is important to note that the Codex Alimentarius [25] has set the Maximum Residue Limit (MRL) for ractopamine in muscle tissue at 10 μg/kg and the acceptable daily intake of no more than 1 μg/kg bw, while for clenbuterol the corresponding levels are significantly lower, namely 0.2 μg/kg and 0.004 μg/kg bw. In some countries, the MRL of CLE is even lower at 0.1 μg/kg [26,27]. These differences highlight the need for their discriminating control. Prior to this study, test systems for CLE with such a selectivity spectrum have not been offered.

The specified state of affairs above integrates different demands, including the prohibition of CLE use, which creates a necessity for new analytical techniques for screening and controlling CLE content. The necessity of high sensitivity of such tests is defined by two main reasons: more stringent confirmation of compliance with the ban on the use of clenbuterol and providing more reliable testing due to higher sample dilution for the elimination of the matrix effects.

When choosing the LFIA format, we focused on using gold nanoparticles (GNPs) as a label. GNPs are most often utilized in LFIAs and do not require additional development for scalable technological production of test systems. The proposed assay does not include signal amplification stages, since they increase the labor intensity and the assay duration. GNPs allow for visual and easy photometrical recording of the assay results, enabling tests to be conducted outside the laboratory. To ensure high sensitivity, our development combined the indirect labeling approach, previously proposed for effective detection of low-molecular-weight toxicants [28], with careful multifactorial optimization of parameters that influence the analytical characteristics of LFIA [29]. These parameters include concentrations of immunoreagents, composition, pH, and ionic strength of the working buffer, used detergent, and organic additives. The study also included testing the developed LFIA with an assessment of its analytical parameters, selectivity, and efficiency of CLE control in meat products, as well as a comparison of the achieved characteristics with the developments of predecessors.

## 2. Materials and Methods

### 2.1. Chemicals

Chloroauric acid, bovine serum albumin (BSA), sodium azide, sodium citrate, dimethyl sulfoxide (DMSO), Triton X-100, Tween-20, and sucrose were purchased from Sigma Chemicals (St. Louis, MO, USA). HEPES (4-(2-hydroxyethyl)-1-piperazineethanesulfonic acid) was from Suzhou Yacoo Science (Suzhou, China). MES 1-hyrate for buffer solutions was from Applichem (Darmstadt, Germany). Goat antibodies against mouse immunoglobulins (GAMI) and donkey antibodies against goat immunoglobulins (DAGI) were obtained from Arista Biologicals (Allentown, PA, USA). Conjugate of GAMI and horseradish peroxidase (GAMI–HRP) was purchased from Servicebio (Wuhan, China). Substrate for peroxidase activity detection based on 3,3′,5,5′-tetramethylbenzidine (TMB) was from Immunotech (Moscow, Russia). Clenbuterol, fenoterol, mabuterol, orciprenaline, ractopamine, salbutamol, terbutaline, ampicillin, chloramphenicol, lincomycin, and tetracycline from Sigma Chemicals (St. Louis, MO, USA) were taken for cross-reactivity testing. All other reagents with analytical or higher purity grades were from Khimmed (Moscow, Russia).

### 2.2. Preparation of CLE Immunogen

CLE solid (4.3 mg) was dissolved in ultrapure water (1 mL), followed by the addition of 1 M HCl solution (45 μL) with thorough mixing. The mixture was incubated at 4 °C for 0.5 h. Subsequently, a 30% NaNO_3_ solution (5 μL) was added, and the reaction was stirred at 4 °C for 1 h to obtain the activated solution. Meanwhile, keyhole limpet hemocyanin (KLH) (10 mg) was dissolved in 0.05 M carbonate buffer (CB, 2 mL). The activated solution was added dropwise to the KLH, maintaining the pH at 9.0–10.0 with NaOH. The final preparation was stirred for 4 h in the dark at room temperature (RT).

### 2.3. Preparation of CLE Coating Antigen

CLE solid (2.56 mg) was first dissolved in ultrapure water (1 mL). After adding 1 M HCl solution (27 μL) and thorough mixing, the solution was incubated at 4 °C for 0.5 h. The activation was initiated by adding a 30% NaNO_3_ solution (3 μL) with subsequent stirring in an ice bath at 4 °C for 1 h. Meanwhile, BSA solid (10 mg) was dissolved in CB buffer (2 mL). The activated CLE solution was then slowly added to the BSA, adjusting the pH to 9.0–10.0. The reaction proceeded for 6–8 h in the dark at RT. The reaction products were transferred into pre-sterilized dialysis bags for dialysis in 0.01 M K-phosphate buffer containing 0.1 M NaCl, pH 7.4. The dialyzed solutions were aliquoted for storage at −20 °C before use.

### 2.4. Animal Immunization and Antibody Production

The animal immunization and antibody production were referred to the previous report [30]. Briefly, the CLE-KLH immunogen was emulsified with complete Freund’s adjuvant and administered to three mice (BALB/c female, 6–8 weeks old) by multiple subcutaneous injections on the back. The primary immunization dose was set at 100 μg of immunogen per mouse. For booster immunizations, incomplete Freund’s adjuvant was used for emulsification, with the first booster dose at 50 μg of immunogen per mouse, followed by three additional boosters at the same dosage. Serum antibody titers against the coating antigen and inhibition rate against CLE were tested using indirect competitive enzyme-linked immunosorbent assay (ic-ELISA). After five immunizations, the mouse demonstrating the highest serum titer and inhibition rate was selected for cell fusion. All animal experiments strictly complied with the “Regulation for the Administration of Affairs Concerning Experimental Animals” in China and were approved by the Animal Ethics Committee of Jiangnan University (approval code: JN. No20240630b2400118[330], approval date: 30 June 2024).

Splenocytes from the selected mouse were fused with cultured SP2/0 myeloma cells using PEG-2000. The resulting hybridomas were further screened by ic-ELISA and subsequently subjected to three rounds of subcloning by limiting dilution to isolate stable monoclonal cell lines secreting CLE-specific antibodies. The screened hybridoma clone (10D2) was expanded in culture, and the mAb was purified from the supernatant using protein G affinity chromatography. The purified mAb was aliquoted and stored at −20 °C before use.

### 2.5. ELISA of CLE

ELISA was implemented in Costar 9018 96-well transparent polystyrene microplates (Corning Costar, Tewksbury, MA, USA). The CLE–BSA conjugate (100 μL, 1 μg/mL) in 50 mM K-phosphate buffer with 0.1 M NaCl, pH 7.4 (PBS) was adsorbed in the wells of a microplate at 4 °C overnight. The microplate was washed fourfold with PBS containing 0.05% Triton X-100 (PBST). Then, CLE solutions in PBST were placed in the wells (50 μL, with concentrations ranging from 100 ng/mL to 10 pg/mL), followed by the addition of anti-CLE antibodies (50 μL, 100 ng/mL in PBST) to each well. The microplate was incubated for 1 h at 37 °C and was fourfold washed with PBST. After that, GAMI–HRP conjugate was added to the wells (100 μL, 1:5000 dilution in PBST), and the microplate was incubated for 1 h at 37 °C. The catalytic activity of the bound HRP was recorded after washing the wells using chromogenic TMB-based substrate (100 μL per well). The optical density (OD) of the product of TMB oxidation at 450 nm was recorded with a Zenyth 3100 microplate photometer (Anthos Labtec Instruments, Salzburg, Austria).

### 2.6. Preparation and Characterization of Gold Nanoparticles

Gold nanoparticles (GNPs) with an average diameter of 30 nm were synthesized as described in [31]. Briefly, a water solution of HAuCl_4_ (1.0 mL, 1%) was added to 97.5 mL of water. The mixture was heated to reflux, and 1.5 mL of 1% sodium citrate solution was added. After 30 min of refluxing the preparation, it was cooled and then stored at 4 °C.

Transmission electron microscopy (TEM) of the obtained GNP preparation was performed on a JEM-100C (JEOL, Tokyo, Japan) microscope at an accelerating voltage of 80 kV and 66,000–100,000 magnification [32]. The size of the GNPs was also characterized by dynamic light scattering (DLS) using a Zetasizer Nano ZS 90 instrument (Malvern Panalytical, Malvern, UK).

### 2.7. Immobilization of Antibodies on GNPs

Before conjugation with GNPs, GAMI were dialyzed against a 10 mM Tris–HCl buffer, pH 8.5. The pH of the GNP solution was adjusted to 8.7 with K_2_CO_3_, followed by the addition of GAMI (6 mg per 1 mL of the GNP solution) [32]. The reaction mixture was stirred for 45 min at RT, and then, a 10% aqueous BSA solution (*w*/*v*) was added to the final BSA concentration of 0.25% and stirred for 15 min. The GAMI–GNP conjugate was separated by centrifugation at 13,400× *g* for 15 min at 4 °C. The obtained residue was dissolved in 10 mM Tris–HCl buffer, pH 8.5, which also contained BSA (1%), sucrose (1%), and NaN_3_ (0.05%) (all *w*/*v*). The obtained conjugate was stored at 4 °C.

### 2.8. Production of Immunochromatographic Test Strips

A CNPC-12 working nitrocellulose membrane with a 15 µm pore size and an AP045 adsorption pad (both from Advanced Microdevices, Ambala Cantt, India) were used for test strip production. CLE-BSA (0.5 mg/mL) and DAGI (0.25 mg/mL) in PBS were immobilized on the working membrane (0.1 μL/mm) to form a test line (TL) and a control line (CL), respectively, by the Iso-Flow dispenser (Imagene Technology, Hanover, NH, USA). The prepared sheets were dried for 24 h at RT, and after that, cut into individual test strips with a 3.0 mm width using an automatic guillotine (KinBio, Shanghai, China). Thus, the quantities of the reactants applied to one prepared test strip were 0.15 μg for CLE-BSA and 0.075 μg for DAGI. Length of test strips was 50 mm. Finally, the produced test strips were placed in packages with silica gel, sealed, and stored at RT.

### 2.9. Preparation of Spiked Meat Samples

Meat samples were purchased from the local market. The samples (2 g) were homogenized and spiked with a CLE standard solution (10 ng/mL) to achieve the contamination levels of 0.25, 0.5, and 1 ng/g. After thorough mixing, the samples were processed according to the recommendations of the ELISA kit Ridascreen Clenbuterol [33]. Specifically, acetonitrile (6 mL) was added to the spiked meat samples. After vigorous stirring for 10 s, the mixtures were stirred upside down for 15 min. The extracts were then centrifuged at 3000× *g* for 10 min. Supernatants (4 mL) were evaporated to dryness under air flow at 50 °C. The obtained residue was resuspended in 2 mL of PBST.

### 2.10. LFIA of CLE

A tested sample (50 μL), specific MAb (8.4 μg/mL, 1 μL), and GAMI–GNPs (OD520 = 1.0, 1 μL) were mixed and incubated for 1 min at room temperature. Next, the test strips were immersed in the mixture and incubated for 14 min. Then, test strips were taken off and scanned using a Canon 9000 F Mark II scanner (Canon, Tokyo, Japan) with a resolution of 600 dpi. The images were analyzed by TotalLab software, version 3.00 (TotalLab, Newcastle Upon Tyne, UK). For statistical processing, all the measurements were performed in triplicate.

### 2.11. Registration and Processing of ELISA and LFIA Data

The dependencies of OD_450_ (for the ELISA) or TL coloration intensity (for the LFIA) (y) on the CLE concentration (x) were approximated using the Origin 9.0 software (OriginLab Corporation, Northampton, MA, USA) by the following four-parametric sigmoidal function:

*y* = (*a* – *b*)/[1 + (*x*/*c*)*_d_*] + *b*,

where *a* is the maximal signal, *b* is the minimal signal, *c* (or IC_50_) is the CLE concentration at which the decrease in y was half maximum, and *d* is the slope of the approximating dependency at point *c*.

The range of CLE concentrations corresponding to the signal decrease from 20% to 80% was evaluated as the working range of the assay. The instrumental LOD (IC_10_) was regarded as the CLE concentration causing a 10% decrease in the colorimetric signal [34]. The visual LOD (cutoff) of the LFIA was expressed as the minimum CLE content causing the disappearance of the TL coloration estimated by the naked eye.

### 2.12. Study of LFIA Cross-Reactivity and Recovery

Cross-reactivities (CRs) of LFIA to other compounds (analogs) were determined as follows:

CR = *IC*_50CLE_/*IC*_50analog_ × 100%,

where *IC*_50_ is the concentration of the compound that leads to a 50% decrease in the registered signal.

The recovery (*R*, %) for spiked samples testing was calculated as follows:

*R* = *x*_theor_/*x*_exp_ × 100,

where *x_theor_* is the spiking CLE concentration and *x_exp_* is the CLE concentration assessed fir the sample testing via the calibration curve.

## 3. Results and Discussion

### 3.1. Immunoreactants Characterization

**Figure 1:**
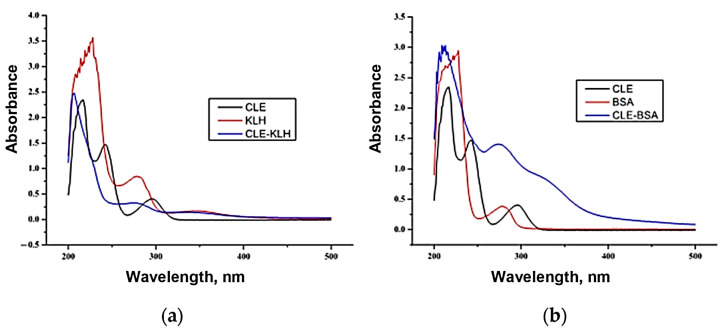
UV-Vis spectroscopy of antigens. (**a**) Spectra of CLE, KLH protein, and CLE-KLH immunogen. (**b**) Spectra of CLE, BSA protein, and CLE-BSA coating antigen.

The synthesized antigens were characterized by UV-Vis spectroscopy. Figure 1a presents the CLE immunogen. During the diazotization synthesis process, diazo bonds were formed, and these bonds had an ultraviolet absorption peak around 350 nm. In Figure 1a, an absorption peak at 350 nm can be clearly observed, confirming the formation of diazo bonds. The characteristic UV absorption peak of the KLH protein is at 280 nm, while that of the CLE-KLH conjugate showed a slight blue shift, indicating the successful conjugation of the CLE-KLH immunogen. Figure 1b displayed the CLE coating antigen. Drawing on the insights from Figure 1a, the UV absorption peak at the position of the diazo bond of the coating antigen was quite distinct. Compared with the characteristic peak of the BSA protein, it showed a slight shift. Moreover, UV absorption peak superposition occurs. All these findings demonstrate that the CLE-BSA coating antigen was also successfully conjugated.

### 3.2. ELISA of CLE

**Figure 2:**
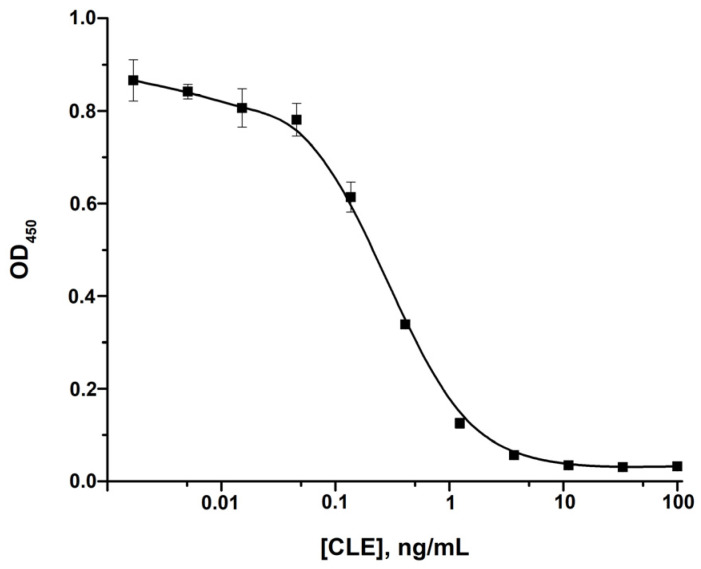
Calibration curve of CLE detection in the ELISA (*n* = 3).

Anti-CLE MAb was utilized for CLE detection by the ELISA. Following the optimal ELISA conditions, namely, a CLE-BSA conjugate concentration of 1 μg/mL and a MAb concentration of 100 ng/mL, CLE was detected with a limit of detection (LOD) of 0.05 ng/mL (Figure 2). Thus, the obtained immunoreactants are suitable for the sensitive detection of CLE and were used in further developments.

### 3.3. Synthesis and Characterization of GNPs and Their Conjugation with Antibodies

**Figure 3:**
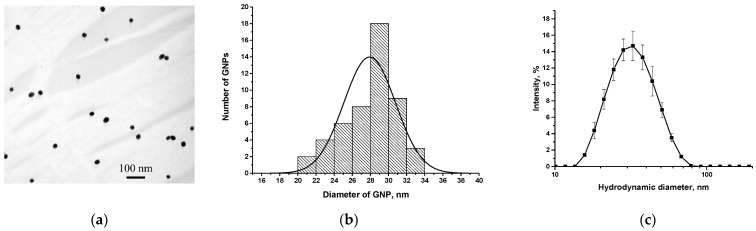
TEM image of the synthesized GNPs (**a**), histogram of their diameter distribution by TEM data (**b**), and distribution of their hydrodynamic diameters by DLS data (**c**).

To ensure a stable and reproducible analytical signal visible to the naked eye, GNPs were used as a label. GNPs were obtained by the citrate reduction method [31]. TEM characterization showed that GNPs did not aggregate (Figure 3a,b). The average diameter of GNPs was 27.9 ± 3.1 nm; the ellipticity (1.25 ± 0.18) confirmed that the particles’ shape was close to spherical. The size of the GNPs was also characterized by DLS. In this case, the average diameter of the GNP was found to be 34 nm (Figure 3c), being somewhat higher as compared with TEM measurements due to the contribution of water molecule shells to the results of DLS measurements.

GNPs were conjugated with anti-species antibodies (GAMI) by physical adsorption. GAMI was used for the conjugation at a concentration of 6 μg/mL following the previously selected optimal protocol, which ensured the mono-layer coverage and stability of the resulting conjugate [32].

### 3.4. Development of the LFIA

**Figure 4:**
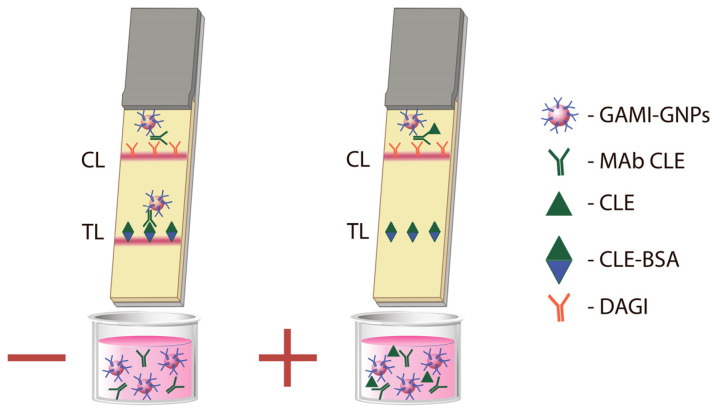
Scheme of the realized LFIA for CLE.

The CLE-BSA conjugate was applied to the working membrane of the test strip to form a TL, and DAGI was adsorbed to make a CL (Figure 4). The GNP-labeled anti-species antibodies were mixed with specific anti-CLE MAb and the tested sample, and the test strip was immersed in this reaction mixture. If there were no CLE in the sample, the complex consisting of MAbs and GAMI-GNPs bound to the CLE-BSA conjugate in the TL would lead to its coloration. The presence of CLE in the sample prevented the binding of the colored complex to CLE-BSA in TL because CLE blocked the antigen-binding sites of MAb. This resulted in the absence of TL coloration.

The CNPC12 nitrocellulose membrane with a pore size of 15 μm was used as the working membrane, which was found to be effective when working with meat matrices [35]. When developing the assay, we used a short test strip (cut to the lower edge of the working membrane), which did not contain a membrane pad. This configuration allowed for a twofold reduction in sample volume and immunoreagents’ consumption. To improve the LFIA characteristics, several key parameters were optimized, including the concentrations of the CLE-BSA conjugate and MAb, the type of working buffer, the concentration of detergents, the ionic strength and pH of the reaction medium, and the presence of additional organic components. To select the optimal combination of variable parameters, the impact of each parameter on the assay sensitivity and the intensity of the TL coloration was estimated. For convenient comparison of the obtained results, a coefficient (K) reflecting the ratio of the TL coloration intensity at the zero point (sample without CLE) and CLE concentration of 0.1 ng/mL was employed.

**Figure 5:**
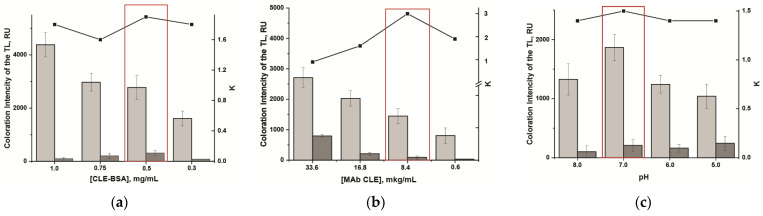
Dependencies of the TL coloration intensity and K value: (**a**) on the concentrations of CLE–BSA in the TL, where the MAb concentration was 11.2 µg/mL; (**b**) on MAb concentrations, where the CLE–BSA concentration was 0.5 mg/mL; (**c**) on pH value of the buffer, where the CLE–BSA concentration was 0.5 mg/mL. The red rectangle highlights the optimal conditions for obtaining high TL coloration intensity and maximum K value.

*Selection of CLE–BSA concentration*. The dependency of TL coloration intensity on the CLE-BSA concentration was studied using MAb at a concentration of 11.2 µg/mL. A reduction in the CLE-BSA amount from 1 to 0.3 mg/mL led to a decrease in the IC_10_ from 0.05 to 0.03 ng/mL; the intensity of the analytical signal declined threefold. A CLE-BSA concentration of 0.5 mg/mL was selected, which ensured an optimal combination of sensitivity, TL coloration intensity, and the maximum K value (Figure 5a).

*Selection of anti-CLE MAb concentration*. The concentration of specific antibodies had a significant effect on the sensitivity of the competitive detection. Reducing the concentration of MAb in the reaction mixture from 33.6 to 0.6 μg/mL allowed decreasing the IC_10_ from 0.3 to 0.02 ng/mL while the cutoff remained unchanged (1 ng/mL). However, upon using MAb at a concentration of 0.6 μg/mL, a significant decrease in the TL coloration intensity was observed, which hampered the evaluation of the results. Therefore, a MAb concentration of 8.4 μg/mL was selected for further experiments (Figure 5b).

*Selection of the working buffer composition*. The optimizations described above were performed in PBST. To assess the effect of the buffer composition on the LFIA performance, two other media were tested, namely, 0.01 M MES, pH 7.7, and 0.01 M HEPES, pH 7.4. In both of them, Triton X-100 and Tween 20 were used as detergents at concentrations of 0.05 and 1%. When replacing PBST with MES or HEPES, a more uniform movement of the liquid front along the test strip was observed. Replacing Triton X-100 with Tween 20 was accompanied by a similar effect. However, performing the LFIA in MES containing Triton X-100 or Tween 20 led to a decrease in the analytical signal and a threefold reduction in the assay sensitivity. When performing the analysis in HEPES containing Triton X-100 or Tween 20, an almost twofold decrease in the intensity of TL coloration was observed compared to PBST. Increasing the concentration of the detergent from 0.05 to 1% led to an additional insignificant reduction in the TL coloration (about 15%). A similar result was obtained by increasing the concentration of Triton X-100 to 1% in PBST. It was shown that when performing LFIA in HEPES containing 0.05% Tween 20, the LFIA sensitivity was comparable to that achieved when using PBST. However, the greater amplitude of the analytical signal in the assay in PBST with 0.05% Triton X-100 made this option preferable.

It was experimentally established that 15 min were required for the sample front to pass through the analytical zones in PBST and for the development of a uniform coloration there. Reducing the testing duration was considered inappropriate because insufficient incubation of the test strips and the sample led to an irregular peak-shaped liquid movement and, accordingly, an incorrect assessment of the TL coloration.

*Selection of ionic strength and pH*. To evaluate the effect of the pH of the reaction medium on the LFIA results, four variants of PBST with pH 5, 6, 7, and 8 were used. It was shown that PBST with pH 7 demonstrated the best results in terms of TL color intensity and LFIA sensitivity (Figure 5c).

It was shown that the absence of NaCl in the PBST led to a 1.5-fold increase in the intensity of TL coloration, and the NaCl concentration of 0.5 M reduced the signal amplitude by 2-fold compared to the common PBST. At the same time, the assay sensitivity in the absence of NaCl and when using 0.5 M NaCl decreases almost twofold. Therefore, the common PBST (50 mM K-phosphate buffer, pH 7.4, containing 0.1 M NaCl and 0.05% Triton X-100) was chosen as the working buffer.

*Effects of organic components on the LFIA.* An important stage of any LFIA is the analyte detection in real samples. Matrix components can have a significant effect on the analytical characteristics of the analysis. Methanol and acetonitrile are often used to extract low-molecular-weight analytes, including CLE, from the analyzed samples [36,37]. So, in this study, the effect of these compounds on the LFIA performance was also assessed. For this, methanol and acetonitrile were added to the working buffer at concentrations of 1, 5, 15, and 25%. It was shown that methanol at concentrations of 1 and 5% slightly increased the amplitude of the analytical signal in the TL compared to that measured after the PBST-based detection. A further increase in the methanol concentration caused a slowdown in the liquid movement and a gradual decrease in the intensity of the TL coloration. The LFIA sensitivity was maintained in these conditions. Thus, methanol at a concentration of 1–15% did not have a significant effect on the analytical characteristics of the LFIA. The addition of acetonitrile to the working buffer resulted in a notable deceleration in the sample movement. At an acetonitrile concentration of 15%, the colored sample was stacked at the bottom of the membrane, and the analysis was impossible. Addition of BSA to the working buffer at a concentration range of 0.025–0.5% resulted in more uniform coloration of test zones, but the sensitivity decreased in these cases.

**Figure 6:**
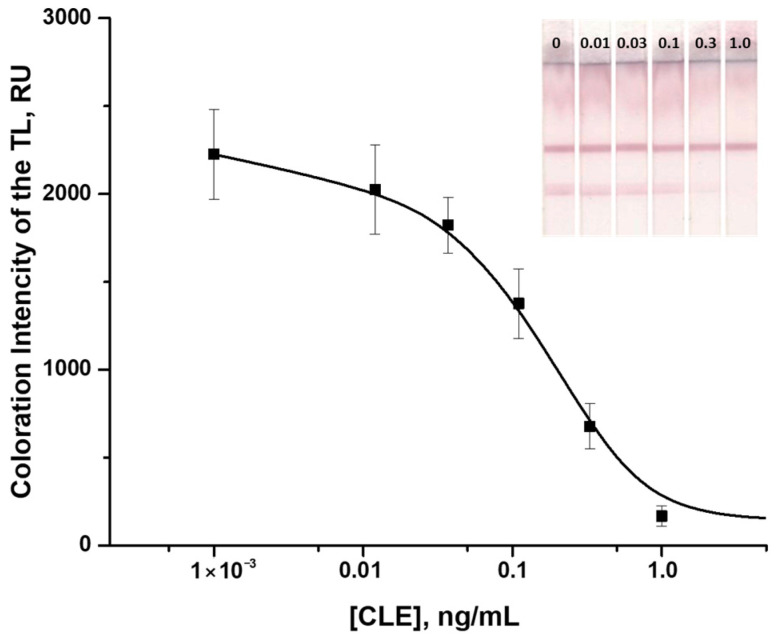
Calibration curve of CLE in the LFIA and images of the corresponding test strips. The concentrations of CLE are as follows: 0 (1), 0.05 (2), 0.1 (3), 0.5 (4), 1 (5), 5 (6), 10 (7), and 100 (8) ng/mL (*n* = 3).

Therefore, the optimal combination of TL coloration intensity and the assay sensitivity was achieved when performing the LFIA in PBST with CLE–BSA and MAb at concentrations of 0.5 mg/mL and 8.4 µg/mL, respectively. The resulting calibration curve was characterized by an instrumental CLE LOD of 0.02 ng/mL, and a working range of detectable concentrations of 0.04–0.90 ng/mL (Figure 6). The cutoff was 0.5 ng/mL, and the assay duration was 15 min.

### 3.5. Specificity of the Developed LFIA

The developed test system was characterized by high cross-reactivities with salbutamol (65%) and mabuterol (36%). Cross-reactivity with terbutaline was 0.5%. The developed system did not yield cross-reactions with other β-agonists, namely ractopamine, orciprenaline, and fenoterol. Veterinary drugs of other classes (ampicillin, chloramphenicol, lincomycin, and tetracycline) did not inhibit the coloration at concentrations up to 10 mg/mL.

As can be seen from Table 1, the molecules of clenbuterol, salbutamol, mabuterol, and terbutaline have the same structure of 2-(tert-butylamino)ethanol at the first carbon atom in the benzene ring. At the same time, the cross-reactivity of these compounds varies from 100 to 0.5%. This may indicate a critical role of the benzene ring for the immune recognition, as well as the nature and location of the substituents at carbon atoms 3, 4, and 5 (for the structures given in Table 1, the numbering corresponds to clockwise movement in the benzene ring, starting with the atom with the longest attached group).

According to the obtained charge distributions, the most potent binding to antibodies is observed in structures with alternating charges on carbon atoms 3, 4, and 5 of the benzene ring in the minus–plus–minus order. The most similar electron density distributions are observed in the clenbuterol–salbutamol–mabuterol group, which exhibits the highest cross-reactivity. However, significant differences in binding within the group indicate that charge distribution within the benzene ring is not the only factor. It can be hypothesized that side substituents at carbon atoms in the benzene ring also influence antibody binding. Thus, a comparison of clenbuterol and mabuterol shows that replacing a single substituent at carbon atom 3 (according to the numbering used) changes cross-reactivity by almost threefold.

### 3.6. Determination of CLE in Meat Samples

**Figure 7:**
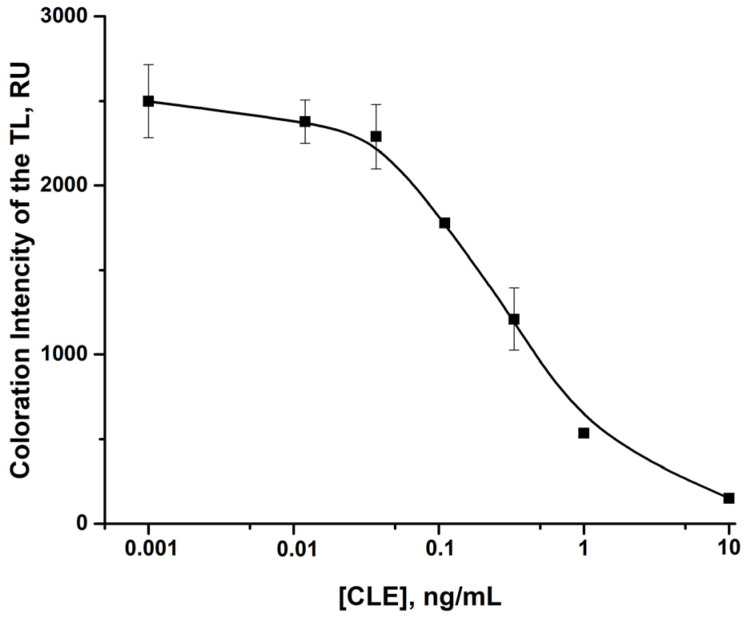
Calibration curve of LFIA for CLE in the meat extracts. The concentrations of CLE are 0, 0.05, 0.1, 0.5, 1, 5, 10, and 100 ng/mL (*n* = 3).

Based on the results of the optimization studies presented above, test strips were manufactured, and the LFIA of CLE in pork extracts was conducted. For meat sample preparation, the technique of the Ridascreen ELISA kit was used. This technique involved analyte extraction, removal of large matrix components, and conversion of the analyte into a matrix compatible with the subsequent immune testing. The CLE-free extract was used to make the calibration curve. It was spiked with CLE, and the LFIA was carried out. Because meat extract is a complex multicomponent matrix, the LFIA efficiency was slightly lower in the obtained extract than that observed in the buffer. According to the calibration curve, the instrumental (photometric) LOD was 0.025 ng/mL, and a cutoff for visual estimation was 1.0 ng/mL (Figure 7).

To verify the efficiency of the developed LFIA, homogenized meat samples were spiked with known amounts of CLE (0.25, 0.5, and 1.0 ng/g). The resulting samples were processed as described in Section 2.9 and tested. The obtained recovery values indicate that the test system can detect 86–104% of CLE in meat samples (Table 2).

### 3.7. Comparison of the Developed LFIA with Other LFIAs

Table 3 compares the analytical parameters of the developed LFIA with those of previously described LFIAs. An analysis of existing developments showed that an increase in the sensitivity of CLE detection is possible, firstly, through the use of new labels. The second way to increase assay sensitivity is to use complex detectors, such as SERS, which allow for a significant gain in sensitivity but make the analysis time-consuming and expensive. In this regard, the GNP-based assay we developed allows for the detection of CLE with high sensitivity, eliminating the need for new labels and complex, expensive methods for their detection. This is an advantage over the mentioned results in the row of tests adopted for on-site use (group A in Table 3). Thus, the developed test system is convenient, simple, and cost-effective.

## 4. Conclusions

An LFIA of CLE and structurally similar β2-agonists using GNPs as labels was developed. Under optimized conditions, LFIA allowed for the CLE detection at concentrations down to 1.0 ng/mL in qualitative mode (visual assessment) and down to 0.02 ng/mL in quantitative mode (photometric recording of color intensity). The assay duration was 15 min. The assay provides integral screening control of CLE and structurally close β2-agonists–salbutamol and mabuterol, distinguishing them from other β-agonists, including β1-agonist ractopamine. The test strips were used to detect CLE in meat samples; a high degree of the analyte recovery (86–104%) was demonstrated. The obtained results display the prospects and competitiveness of the developed method for the rapid and sensitive on-site screening control of priority β2-agonist presence in meat products.

## Figures and Tables

**Table 1:** Cross-reactivity of β-agonists in the developed LFIA.

Compound	Molecular Structure and Charge Distribution *		Cross-Reactivity, %
Clenbuterol	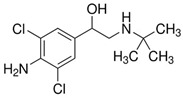	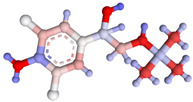	100
Salbutamol	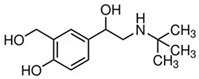	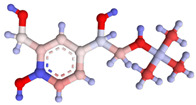	65
Mabuterol	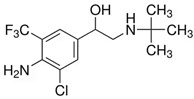	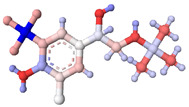	36
Terbutaline	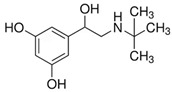	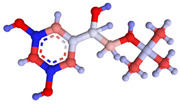	0.5
Orciprenaline	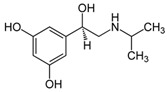	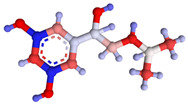	<0.01
Ractopamine	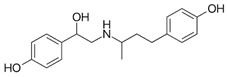	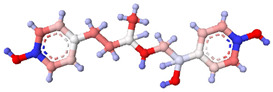	<0.01
Fenoterol	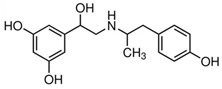	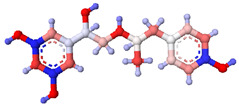	<0.01
		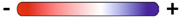	

* The charge distributions were drawn using the Atomic Charge Calculator program (https://webchem.ncbr.muni.cz/Platform/ChargeCalculator/, accessed on 24 October 2025).

**Table 2:** Recoveries of CLE from meat samples (*n* = 3).

Added Content (ng/g)	Revealed Content			Recovery, %
	Average Value, ng/g	Standard Deviation, ng/g	Relative Standard Deviation, %	
0.25	0.26	0.05	19	104
0.5	0.43	0.06	14	86
1.0	0.97	0.06	6	96

**Table 3:** Comparison of the developed and the published LFIAs for clenbuterol.

Detection and Additional Techniques	Labels	Limits of Detection	Cross-Reacting Compounds	Real Samples	Reference
Colorimetry	GNPs	5 ng/mL	Not found	Pork, chicken, sausage	[18]
Colorimetry	Coomassie brilliant blue	2 ng/mL	Not found	Milk, swine liver, pork tenderloin	[38]
Colorimetry	GNPs	0.59 ng/mL	Not found	Beef and pork liver	[19]
Colorimetry	Prussian blue nanoparticles on GNPs	0.27 ng/mL	Not found	Pork	[39]
Colorimetry	Magnetic Prussian blue nanozyme	0.2 ng/mL	Not found	Pork, mutton	[17]
Colorimetry	Natural cuttlefish ink nanoparticles	0.179 ng/mL	Not found	Pork, beef	[22]
Colorimetry	Tannic acid nanospheres	0.13 ng/mL	Not found	Beef and pork liver	[19]
Colorimetry	Bi_2_S_3_ nanoparticles	0.1 ng/mL	Not found	Pork, beef, milk	[21]
Colorimetry	GNPs	0.02 ng/mL	Salbutamol, mabuterol	Pork	This work
Fluorimetry	Prussian blue nanoparticles on GNPs	0.152 ng/mL	Not found	Pork	[39]
Fluorimetry	GNPs and time-resolved fluorescent nanobeads	0.04 ng/mL	Not found	Pork	[16]
Fluorimetry	Fluorescent latex nanoparticles	0.025 ng/mL	Mabuterol, banbuterol, brombuterol, bromchlorbuterol, cimaterol, cimbuterol	Pork	[15]
Photothermal measurements	Natural cuttlefish ink nanoparticles	0.076 ng/mL	Not found	Pork, beef	[22]
Magnetic enrichment + photometry	Magnetic nanoparticles	0.007 ng/mL	37 other β-agonists	Pork, swine urine	[23]
Surface-enhanced Raman scattering	Au/Au core/shell nanostars	0.05 ng/mL	Not found	Pork, chicken, sausage	[18]
Magnetic enrichment + surface-enhanced Raman scattering	Zero-background SERS tags and magnetic nanoparticles	0.00087 ng/mL	Not found	Urine	[40]

## Data Availability

The original contributions of this study are included in this manuscript, and further inquiries can be directed to the corresponding author.
